# Sanitation practices and perceptions in Kakuma refugee camp, Kenya: Comparing the status quo with a novel service-based approach

**DOI:** 10.1371/journal.pone.0180864

**Published:** 2017-07-13

**Authors:** Raymond Nyoka, Andrew D. Foote, Emily Woods, Hana Lokey, Ciara E. O’Reilly, Fred Magumba, Patrick Okello, Eric D. Mintz, Nina Marano, Jamae F. Morris

**Affiliations:** 1 CDC Kenya, Nairobi, Kenya; 2 Sanivation LLC, Naivasha, Kenya; 3 Division of Foodborne, Waterborne, and Environmental Diseases, National Center for Emerging and Zoonotic Infectious Diseases, Centers for Disease Control and Prevention, Atlanta, GA, United States of America; 4 Norwegian Refugee Council, Nairobi, Kenya; 5 African-American Studies, Georgia State University, Atlanta, GA, United States of America; TNO, NETHERLANDS

## Abstract

Globally, an estimated 2.5 billion people lack access to improved sanitation. Unimproved sanitation increases the risk of morbidity and mortality, especially in protracted refugee situations where sanitation is based on pit latrine use. Once the pit is full, waste remains in the pit, necessitating the construction of a new latrine, straining available land and funding resources. A viable, sustainable solution is needed. This study used qualitative and quantitative methods to design, implement, and pilot a novel sanitation system in Kakuma refugee camp, Kenya. An initial round of 12 pre-implementation focus group discussions (FGDs) were conducted with Dinka and Somali residents to understand sanitation practices, perceptions, and needs. FGDs and a supplementary pre-implementation survey informed the development of an innovative sanitation management system that incorporated the provision of urine and liquid-diverting toilets, which separate urine and fecal waste, and a service-based sanitation system that included weekly waste collection. The new system was implemented on a pilot scale for 6 weeks. During the implementation, bi-weekly surveys were administered in each study household to monitor user perceptions and challenges. At the end of the pilot, the sanitation system was assessed using a second round of four post-implementation FGDs. Those who piloted the new sanitation system reported high levels of user satisfaction. Reported benefits included odor reduction, insect/pest reduction, the sitting design, the appropriateness for special populations, and waste collection. However, urine and liquid diversion presented a challenge for users who perform anal washing and for women who had experienced female genital mutilation. Refugee populations are often culturally and ethnically diverse. Using residents’ input to inform the development of sanitation solutions can increase user acceptability and provide opportunities to improve sanitation system designs based on specific needs.

## Introduction

Globally, an estimated 2.5 billion people lack access to improved sanitation.[1] A facility is considered improved when it hygienically separates human excreta from human contact. These facilities include: flush/pour flush to a piped sewer system, septic tank, or pit latrine; ventilated improved pit latrines; pit latrines with slabs; composting toilets. Sanitation facilities shared with other households are not considered to be improved. [1] This is true of more than half of the population in sub-Saharan Africa.[2] Even when improved sanitation is available, in many sub-Saharan countries less than 10% of human waste is properly treated before disposal in the environment.[3][4] Limited access to improved sanitation and improper human waste disposal increases the risk of diarrhea-related morbidity and mortality. With the rapid influx of refugees and constrained sanitation options, conditions in refugee camps are especially challenging.[5][6][7][8] Implementing well-designed sanitation solutions that aim to decrease exposure to human fecal waste can prevent diarrheal disease in refugee camps.[9][10]

Kakuma refugee camp, located in Turkana County in northwestern Kenya, was established in 1992 to temporarily accept approximately 20,000 refugees from Sudan and Ethiopia.[11] At the time of the study, April 2014, the camp population exceeded 150,000 refugees representing 19 different nationalities. As conflict in neighboring countries continues, new refugees have continued to arrive.[12][13] The population growth has strained the existing sanitation system. The Sphere Handbook, which describes the minimum standards needed for affected populations to survive and recover in stable conditions and with dignity, indicates that latrines should be shared by no more than 20 people, and toilets should be located no more than 50 meters from a household.[14] As of 2014, only 90% of camp communal latrines met the minimum standards.[15]

Widely used, pit latrines, a basic form of sanitation comprised of large holes dug into the ground covered by a slab, are regarded as the simplest and cheapest improved sanitation option available.[16] However, pit latrines may not always be the most adequate solution for use in protracted refugee situations, defined by United Nations High Commissioner for Refugees (UNHCR) as “one in which refugees find themselves in a long-standing and intractable state of limbo”.[17] Once the pit latrines are full, waste remains in the pit and a new latrine is constructed in the remaining space near the old one, ultimately stretching scarce land and funding resources—UNHCR and implementing partners provide the supplies for constructing and replacing pit latrines, which has been estimated to be approximately $100 per unit per year in protracted settings similar to Kakuma refugee camp.[18] Further, beyond space, safe waste disposal, and funding considerations, pit latrine use can pose additional challenges for special populations, including children and people who are sick, disabled or elderly. There is a need for other viable, more sustainable sanitation solutions that can accommodate diverse and growing refugee populations, particularly those that are informed by the communities themselves.

Alternative solutions piloted in other refugee settings highlight the potential of urine diverting toilets as a safe and sustainable sanitation solution.[19][20][21] These technologies offer several benefits including the possibility of waste reuse and odor/pest reduction [19] and are marked as particularly appropriate settings in need of an alternative to pit latrines.[21] However, additional considerations, not as well represented in the literature, including the appropriateness for special populations (children, persons with disabilities, and the elderly) and acceptability in settings where camp residents have diverse sanitation practices and preferences must also be included.

In 2013, a consortium of agencies, including the U.S. Centers for Disease Control and Prevention, Sanivation LLC (hereafter referred to as Sanivation), UNHCR, and the Norwegian Refugee Council (NRC)—the implementing partner in charge of sanitation at Kakuma refugee camp—piloted a novel method for sanitation in the camp which would reduce/eliminate the need for pit latrine construction, reduce the number of users per toilet, increase accessibility for special populations, create a system for the safe disposal and possible reuse of human fecal waste, and reflect the self-reported needs and preferences of camp residents. Qualitative and quantitative data were collected to guide the development of an innovative sanitation management system and evaluate user perspectives and preferences.

## Methods

### Study setting

Kakuma refugee camp is located in northwest Kenya. Of the 150,000 camp residents at the time of the study, more than three-quarters were Somali and South Sudanese, representing 41% and 35% of camp residents in 2014, respectively.[13] This pilot was limited to Somali and Dinka residents, from Somalia and South Sudan, respectively, who were older than 18 years old and living in the oldest part of the camp. Due to the limited space available to construct new latrines and the representation of Somali and Dinka residents among the camp population, camp managers identified this area of the camp as having the most urgent and diverse sanitation needs.

### Pre-implementation

#### Participant recruitment and moderator training

NRC led community mobilization and recruitment efforts. Before recruitment, NRC engaged community leaders and members regarding the purpose and scope of this project and explained the overall sampling strategy. A purposive sampling method, centered on gender and language, was used to ensure an adequate number of Somali and Dinka participants. Moderators, who lived in the camp and spoke English and at least one of the FGD languages (Dinka or Somali), led each FGD. In preparation, each moderator participated in a 2-day training session to review FGD techniques and discuss the moderator’s guide.

#### Data collection

**Fig 1:**
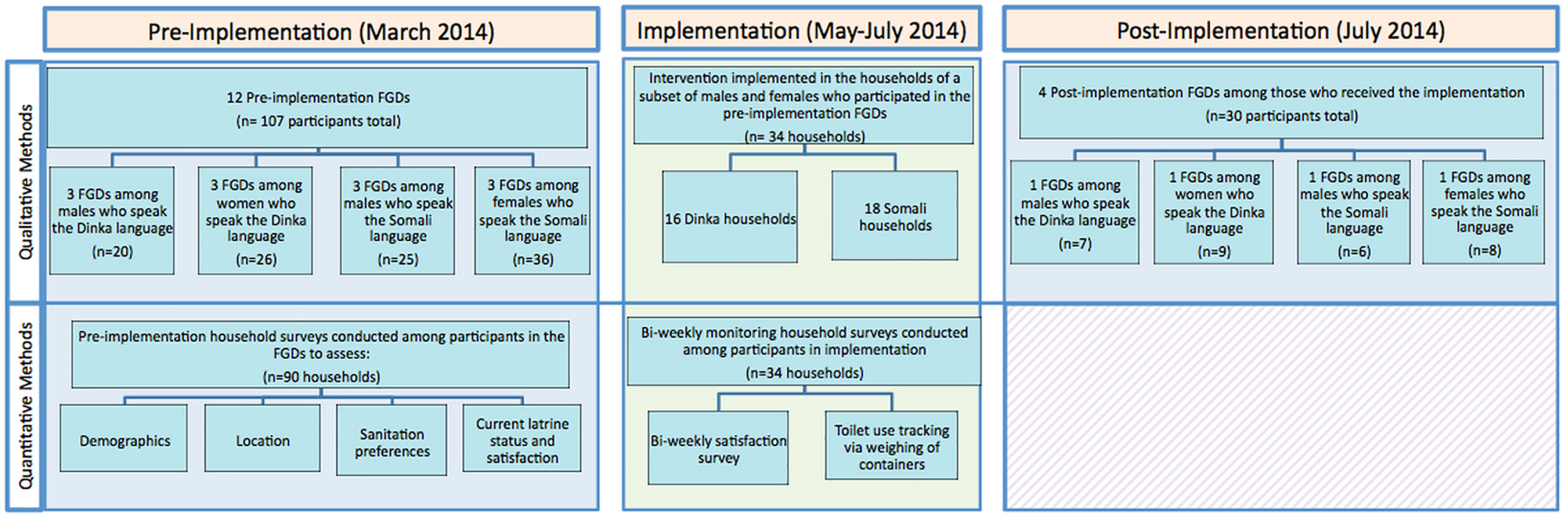
Flow chart outlining qualitative and quantitative methods used.

We conducted 12 pre-implementation FGDs, divided equally by language and gender (Fig 1). Each FGD had 6–10 participants, lasted approximately 90 minutes, and focused on the benefits and challenges of the current sanitation system and ideas for improvements; all FGDs were audio-recorded. Following the initial set of FGDs, participants also completed a pre-implementation survey, which was used to establish an understanding of household demographics and sanitation needs. Sanivation used the pre-implementation FGD data to better understand sanitation challenges in the camp and to inform the design of a complete sanitation system.

### Intervention implementation

#### Intervention selection criteria

Households that participated in the pre-implementation FGDs were asked about their interest in participating in a six-week pilot of a novel sanitation system. Of those interested, 32 households were randomly selected in three geographic clusters, after controlling for a representative sample of households with disabled and/or elderly persons. Two Somali households withdrew from the study in the first week and were replaced. Thus, in total, 16 Somali and 16 Dinka households were selected to pilot the new sanitation system (Fig 1).

#### Intervention description

Sanivation used the data from the pre-implementation FGDs to inform the design of an alternative sanitation system (described below) to include: a regularly serviced toilet unit, solar waste treatment, and the production of charcoal briquettes. The new toilets were designed with two openings, one for urine and other liquids and one for feces, to promote cleaner and more efficient disposal of the waste material.

**Fig 2:**
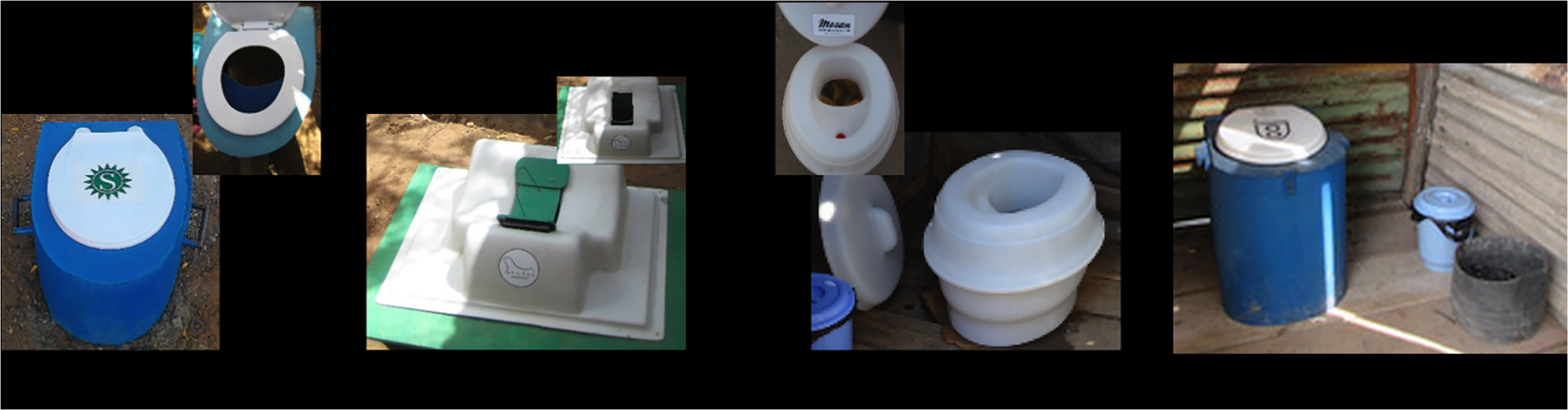
Images of the three urine-diverting, container-based toilets included in the study. (A) Choopoa, (B) SafiChoo, and (C) MoSan; (D) Choopoa.

Each of the 32 participating households received a urine and liquid diverting, container-based toilet (Fig 2), a waste disposal bin for menstrual hygiene products and other nonorganic solid waste, and an ash container to store ashes from cooking to be placed over the feces as a desiccant. Based on the results of pre-intervention FGDs and survey, three container-based toilets with different characteristics, all designed to meet the needs of displaced populations, were selected for the pilot: 1) *MoSan* (Mosan, Zurich, Switzerland), a completely pre-fabricated and highly portable sit toilet; 2) *Choopoa* (Sanivation, Naivasha, Kenya), a locally resourced and fabricated sit toilet; and 3) *SafiChoo* (Wish for Wash, Atlanta, USA), a partially pre-fabricated sit/squat toilet (Fig 2). Household members received an orientation from study staff about how to use the toilet, including guidance on the use of ashes to aid in odor control and instructions to call service representatives if they had questions about the toilet. Trained service representatives visited each household twice per week to collect and replace waste containers. Service representatives transported the filled containers to a central location within the camp, where a locally fabricated solar concentrator, which captures solar energy to thermally inactivate pathogens in fecal waste, rendered the waste material safe for reuse.[22] Treated feces were combined with other waste products, including charcoal dust, to create briquettes for heating or cooking. (For a more extended consideration of the design of the solar concentrator and pathogen inactivation data see http://sanivation.com/). The briquettes were tested for safety and performance, but were not distributed to study households. However, participants were asked about their willingness to use treated waste for fuel. The pilot implementation of the service-based sanitation system was planned as a six-week intervention in Kakuma, after which, the pilot would be assessed to determine suitability for potential scale-up. From the outset, it was envisaged that modifications may be necessary as a result of the pilot, thus, the protocol included decommissioning of the toilets at the end of the pilot period. All study households were made aware of this as part of the informed consent process.

#### Data collection

Throughout the implementation, bi-weekly surveys were conducted at weeks 1, 3, and 5 in each intervention household to facilitate routine monitoring, promote hygiene, and evaluate user satisfaction (Fig 1). If possible, we interviewed both the female and male heads of household; however, at least one member was interviewed. Additionally, fecal containers, collected twice weekly from toilets at each household, were weighed to help track toilet usage. If a household decided to voluntarily withdraw from the intervention, we discontinued bi-weekly surveys and invited an adult household member to participate in the post-implementation FGD.

### Post-implementation

#### Data collection

Following implementation, one representative from each intervention household was assigned to one of four post-implementation FGDs, with 6–10 participants each, organized by gender and language (Fig 1). Topics of discussion included the impact of the intervention on the family’s life, noted challenges, perceptions of the collection process, perspectives on a possible scale-up, and opinions regarding the use of briquettes made from the treated waste.

### Data management and analysis

#### Qualitative data

Certified translators for each language transcribed the audio recordings; the transcripts were then translated into English. Study moderators reviewed the English transcripts to ensure consistency between the audio recording, the original transcript, and the English version.

The moderator’s guide and FGD transcripts were used to develop a codebook organized by theme. NVivo^®^ qualitative data management and analysis software was used to support coding and analysis (NVivo^®^ Version 10, Burlington, M.A.). Transcripts were coded independently by two members of the research team, and codes were compared for inter-rater reliability.[23] No personal identifiers were collected or included in subsequent reports.

#### Quantitative data

Trained surveyors used a combination of hard-copy surveys and an Android mobile platform, Mobenzi^®^, to gather the pre-implementation and bi-weekly survey data. Data were analyzed at the household level. The female head of household’s responses were chosen in instances where both the male and female were interviewed. Likert-scale and demographic data were collected. Given the limited sample size, only frequencies are presented.

### Generalizability

We acknowledge that the sample size and sampling methods used limit the generalizability of the study, and thus, the data presented below can only be assumed to represent the experiences of those who participated. However, the aim of the study was to collect rich, grounded data that reflected the sanitation experiences and perceptions of camp residents to inform the development of a novel sanitation option and to evaluate residents’ experiences with the design. Populations are most familiar with their specific sanitation challenges and needs—this may be particularly true in protracted refugee situations. We firmly believed that eliciting residents’ insight and guidance results in a more culturally sensitive and appropriate system.

### Research ethics

The protocol was approved by the Kenya Medical Research Institute Scientific Steering Committee (SSC # 2737) and the Kenya Medical Research Institute National Ethical Review Committee. Written informed consent was obtained from all participants.

## Results

### Pre-implementation results

#### Pre-implementation participant demographics

A total of 107 camp residents participated in the 12 pre-implementation FGDs. Each FGD had between 6–10 participants and lasted approximately 120 minutes. Slightly more Somali speakers than Dinka speakers, at 57.0% and 43.0%, respectively, and slightly more women than men, at 57.9% and 42.1%, respectively, participated (Table 1). FGD participants had a median age of 27 years (range 18–60) and had lived a median of 7 years in Kakuma (range <1–22). Participants reported a household size of 2–36 persons, with a median of 8 persons. The median number of household members ≤18 years old was five. In total, 61.7% of pre-implementation FGD participants reported owning a latrine (Table 1). Reported latrine ownership among Somali and Dinka ethnic groups was 72.1% and 47.8%, respectively (Table 2).

**Table 1:** Demographic characteristics of participants in the pre- and post-implementation FGDs, Kakuma Refugee Camp, Kenya.

Characteristic	Category	Pre-implementation n = 107	Post-implementation n = 30
Age in years	Median (range)	27 (18–60)	30 (17–66)
Number of years in Kakuma	Median (range)	7 (<1–22)	5 (1–23)
Number of people living in the household	Median (range)	8 (2–36)	10 (1–56)
Number of people in the household <5 years	Median (range)	2 (0–8)	2 (0–10)
Number of people in the household 5–18 years	Median (range)	3 (1–10)	3 (1–17)
Number of people in the household ≥18 years	Median (range)	5 (1–21)	4 (1–33)
Gender	Male, n (%)	45 (42.1%)	13 (43.3%)
	Female, n (%)	62 (57.9%)	17 (56.7%)
Ethnic Group	Dinka, n (%)	46 (43.0%)	16 (53.3%)
	Somali, n (%)	61 (57.0%)	14 (46.7%)
Latrine ownership	Yes, n (%)	66 (61.7%)	22 (75.9%)
	No, n (%)	41 (38.3%)	7 (24.1%)

**Table 2:** Gender and latrine ownership by nationality in the pre- and post-implementation FGDs, Kakuma Refugee Camp, Kenya.

Phase	Characteristic	Category	Dinka participants n = 46 (n %)	Somali participants n = 61 (n %)
Pre-implementation	Gender	Male	20 (43.5%)	25 (41.0%)
		Female	26 (56.5%)	36 (59.0%)
	Latrines	Yes	22 (47.8%)	44 (72.1%)
		No	24 (52.2%)	17 (27.9%)
Post-implementation	Gender	Male	7 (43.8%)	6 (42.9%)
		Female	9 (56.3%)	8 (57.1%)
	Latrines	Yes	9 (56.3%)	13 (100%)
		No	7 (43.8%)	0 (0%)

#### Previous sanitation experiences

Participants described a range of sanitation experiences before their arrival at the camp. Dinka participants, often from rural villages, were more likely to report the previous use of open defecation. Both men and women described going into the forest or the “bush” for urination and defecation. At times, this practice was described as a product of culture or tradition, as indicated by a Dinka male, “…we did it as a cultural way of life.” However, it was most often described as a necessity and attributed to a lack of resources and education. One Dinka woman stated, “People were not educated [in] those days… [w]e could just go and defecate in the forest.”

Unlike Dinka participants who consistently noted similar sanitation patterns, Somali experiences were more varied. Many Somalis reported having consistent access to improved sanitation before arriving in Kakuma. While some detailed living a more nomadic lifestyle that included open defecation, most noted the use of concrete or wooden structures with drop-hole covers or latrines made of brick that channeled to a septic tank. One male participant described the latrines he used before coming to the camp as, “…the normal latrines that are found in mansions and big houses.” According to participants, these latrines could better accommodate individuals with special needs, including pregnant women, individuals with disabilities, and the elderly.

#### Reported benefits of current sanitation in Kakuma

While both groups applauded the cleaning supplies provided by NRC, which aided in latrine cleaning and upkeep, overall perceptions of sanitation at the camp varied primarily according to group membership. For most Dinka participants, sanitation at the camp was generally viewed as a marked improvement. Using latrines was better than open defecation. One Dinka male stated, “[H]ere it looks totally good as compared to going to the bush.” Despite citing a need for additional latrines and more cleaning and construction supplies, Dinka perspectives generally associated latrine use, as compared to open defecation, as a step toward more modern sanitation practices. When referencing sanitation at the camp, a female Dinka participant noted, “Here at Kakuma, we tend to live like people who have gone to school and like rich people, or like very responsible people.” For Somali participants, many of whom reported having used improved sanitation before coming to Kakuma, the current sanitation system was not described as an improvement but rather as a system largely in need of improvements.

In general, Somali men and women recognized that sanitation options at the camp were limited. Latrines were described as affordable and as a better option than open defecation. However, latrines were used because they were provided. One Somali woman offered, “I only like this because I cannot see any other.” Similarly, reflecting the sentiments of a number of Somali participants, one male offered, “It is only better than defecating openly because in open defecation you see the feces.” This theme was present throughout the Somali FGDs, irrespective of gender.

#### Reported challenges of current sanitation situation in Kakuma

When both Dinka and Somali participants noted the challenges of the current system or offered critiques, three broad themes emerged: lack of latrines/overcrowding, latrine cleanliness, and latrine design/construction.

Lack of latrines/Overcrowding. Both Somali and Dinka participants frequently reiterated the need for more latrines, “What we lack is latrines; the ones available are not enough for us” (Dinka male). Some Dinka families with inadequate latrine access sometimes engaged in open defecation near the river bank. “The number of latrines is fewer than the number of users; for example, one latrine might be shared by at least 20 people” (Dinka female). During one of the FDGs, a Dinka woman shared a very telling picture of the magnitude of overcrowding, “[I]n our area, one latrine is shared by 10 households, which consists of adults and children; these get filled up after about 1 month.” Her description was not isolated, but echoed sentiments expressed in all the Dinka pre-implementation FGDs.

Somali participants reported sharing a latrine with an average of 10 family members. However, like Dinka FGD participants, Somalis also discussed the need for more latrines. “We do not like the system, but we have been provided it. One latrine, irrespective of household size, is what is permitted to us. We wish to have two, or even four, latrines” (Somali woman). Overcrowding also influenced participants’ preference for sit versus squat toilets. Households that shared a latrine with fewer than 10 people preferred a sit toilet, while households that shared a latrine with more than 20 people preferred a squat toilet. This was due to hygiene reasons where less people were likely to keep the sit toilet clean than when shared by many.

Latrine Cleanliness. Although participants from all of the FGDs indicated that some cleaning supplies were provided, the need for more cleaning supplies was often cited. Dirty latrines, exacerbated by overfilling, were described as having a foul odor and serving as a breeding ground for insects, namely cockroaches. When describing some of the challenges with his latrine, one Somali male noted the, “unpleasant smell that gets into our house…and… our houses are infested by cockroaches from the latrine, especially from those filled-up ones.” Dinka women and men noted their frustration with practices that contributed to perpetually dirty latrines, such as lack of drop-hole covers, and use of communal, rather than family-owned, latrines, which made it difficult to assign responsibility for cleaning.

Latrine Design/Construction. Though rarely noted in the Dinka FGDs, Somali participants were very concerned about the design of the latrines, which they believed were not well-suited for special populations. According to Somali participants, children were at risk of falling in and the elderly and disabled had difficulty with the lack of a comfortable and safe place to sit. One participant noted, “There are different categories [of] users. They include the aged and disabled, who can’t use these latrines because they don’t have a raised sitting place.” (Somali male). Somali participants also indicated that the latrines were poorly suited for pregnant women and sick individuals.

#### Suggestions for improvements

Participants had a number of clear recommendations for improvements to the sanitation system in the camp, including increasing the number of latrines to better accommodate the growing camp population, consistently providing cleaning supplies to reduce the presence of odor and control pests, and incorporating options more appropriate for special populations a raised seat for special populations.

### Design of the new sanitation system

Informed by the pre-implementation FGDs, Sanivation designed a complete sanitation system that incorporated toilets with service-based waste removal, treatment, and reuse. Toilets (described above) were designed for household-level use and were selected according to the following criteria: considerations for special populations (children, disabled persons, and the elderly), ability to sit while defecating, emptyable containers to prevent foul odors and overfilling, appropriate for the environment in Kakuma, and limit the presence of insects. A total of 32 toilets (8 MoSan, 16 Choopoa, and 8 SafiChoo) were distributed equally among the 16 Somali and 16 Dinka households. If residents already had pit latrines, toilets were placed on top of the pit latrine slab in the existing latrine superstructures. If residents lacked a family latrine, new superstructures were constructed on their plot and new toilets were placed inside. Superstructure construction and toilet installation were completed in 1–5 days. Trained service representatives from the resident population visited each household twice per week to collect and replace containers.

### Results from container weights and bi-weekly surveys

**Fig 3:**
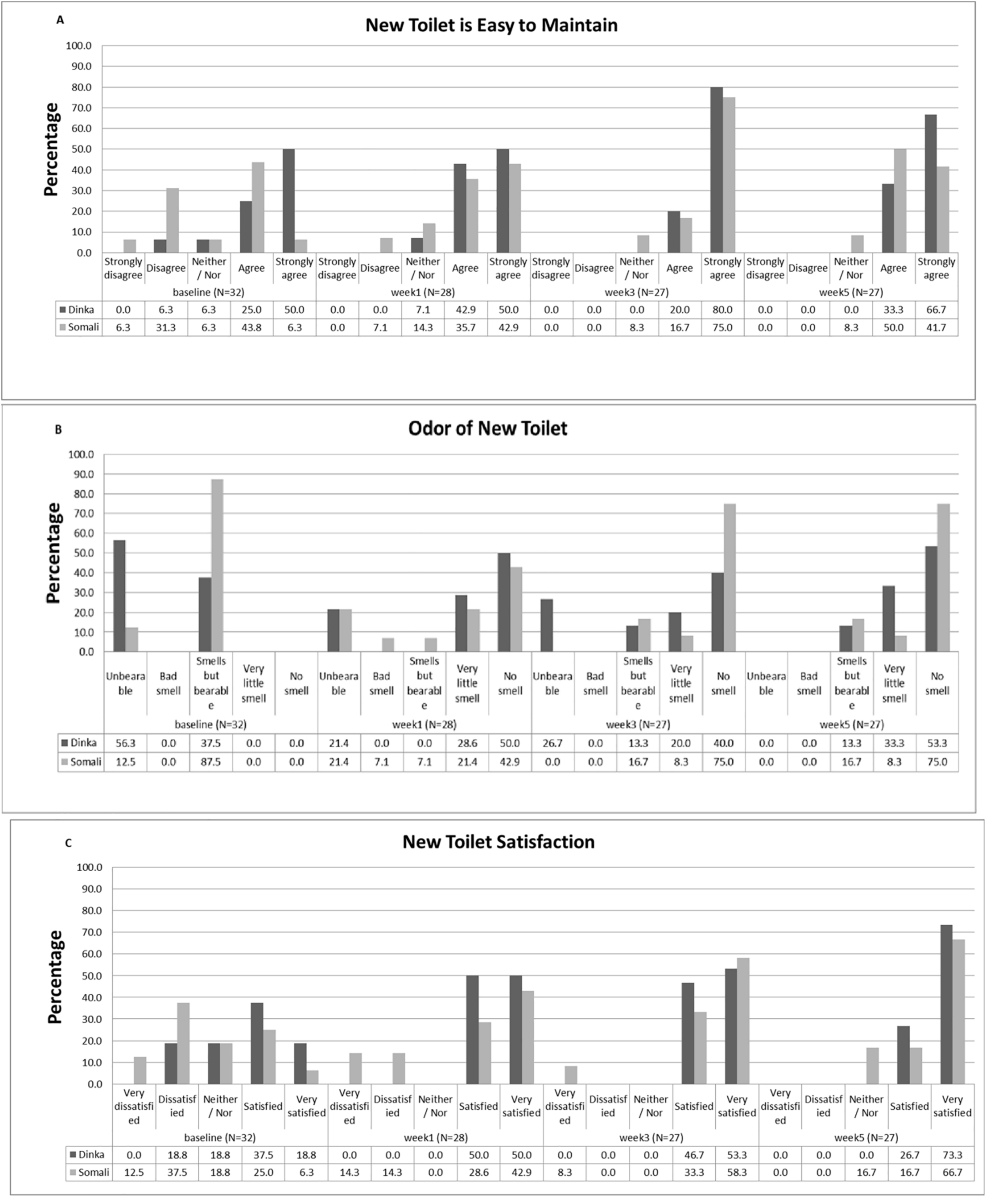
Level of satisfaction with the new toilets as reported in the bi-weekly surveys. (A) How easy it is to maintain the toilet? (B) What is the odor of the toilet? (C) How satisfied are you with your toilet.

Container weights increased throughout the intervention period, from an average of ~0.11 kg per person during week 1 to an average of ~0.23 kg per person during week 5. By the fifth week, 100% of Dinka respondent households and 79% of Somali respondent households were either satisfied or very satisfied with the new toilet, and 85% percent of all households indicated that the toilet had very little to no smell (Fig 3). As a sanitation solution for the elderly and disabled, 100% of Dinka household respondents and 92% of Somali household respondents preferred the new system by the fifth week (Table 3). During the course of the pilot, a total of four Somali households and one Dinka household stopped participating in the intervention due to a preference for pit latrines (4) or moving from the refugee camp (1).

**Table 3:** Percentage of respondents, by demographic group, who reported, in the bi-weekly Somali surveys, that they preferred the new toilets, Kakuma Refugee Camp, Kenya.

	Week 1 (n = 30)		Week 3 (n = 26)		Week 5 (n = 27)	
Group^†^	Dinka	Somali	Dinka	Somali	Dinka	Somali
Respondent preferred the new toilet	85.7%	50.0%	100.0%	83.3%	93.3%	69.2%
Respondent reported elderly/disabled persons preferred the new toilet	92.9%	100.0%	100.0%	100.0%	100.0%	92.3%

^†^Respondents were asked about the preference for themselves and also about the preference of the toilet among elderly and disabled groups.

### Post-implementation results

#### Post-implementation participant demographics

A total of 30 residents participated in the four post-implementation FGDs (Fig 1). Each FGD had between 6–10 participants and lasted approximately 90 minutes. There were slightly more Dinka participants than Somali, at 53% and 47%, respectively, and slightly more women than men participated, at 57% and 43%, respectively. FGD participants were an average of 30 years old (range 17–66) and had spent a median of 5 years in Kakuma (range <1–23). Participants reported an average of 10 persons per household (Table 1).

#### Reported benefits of the new system

Participants noted several benefits associated with the use of the new system. The benefits described were strikingly similar across all groups and included: the reduction of any foul odor, reduction of insects/pests, the sitting design of the toilets, the appropriateness of the new toilets for special populations, and waste collection.

Reduced Smell and Reduced Pests. Attributed to both the waste collection and the use of ashes, participants noted that the new toilets did not smell, either at all or considerably less than the previous system. During one of the FGDs, a Dinka male shared, “We have realized that we were given very good [toilets]. I like using it because of lack of foul smell.” Participants also reported a reduction in the presence of roaches and other pests.

Sitting. The sitting feature was cited as a positive attribute in all of the post-implementation FGDs. Participants often spoke specifically about their own experience. Reflecting sentiments shared in all of the FGDs, one Dinka female indicated, “I like it because there is no more squatting. My knees ache no more because of squatting.” Similarly, a Somali male shared, “It gave us a relaxing position that makes one feel more comfort compared to the other latrines that we were using before.” The raised seat was also noted as extremely important and appropriate for elderly persons and people with disabilities. Instead of squatting or sitting on the slab, the new toilets allowed them to sit more comfortably. In general, the new toilets were also thought to be appropriate for most children, as they reduced the potential for falling in.

Waste collection. In all post-implementation FGDs, participant responses to waste collection were overwhelmingly positive. Men and women from both language groups described feeling thankful and cared for when the waste was removed. While discussing waste collection in the FGD, one Somali male stated, “I like the fact that we are being cared for and that, in a week, the container is replaced twice” (Somali male). Waste collection was viewed as healthy, limiting the accumulation of waste, a point of critique for the previous system. Participants preferred for their waste to be collected by someone else, and expressed discomfort at the possibility of carrying and discarding their own waste. Reflecting the sentiments of many FGD participants, one Somali male noted, “I feel good when [my] container… is replaced… I am also happy that someone else comes to collect it.”

#### Reported challenges of new sanitation system

Participants were asked to discuss any challenges associated with use of the new system. Several challenges were noted, and ranged from perspectives on the toilet design to user experiences. Both common and group-specific challenges were considered. The most common challenges noted by both groups were toilet height and lack of roof.

Toilet Height. Although the toilet was considered appropriate for certain special populations, particularly elderly persons and those with a disability, there was some disagreement about the appropriateness of the toilets for very young children, particularly those younger than 3 years of age. According to the participants, the sitting basin was too high, at times posing a challenge for younger children.

No Roofs. The absence of a roof was noted as a challenge. Participants indicated that direct sun exposure made the toilet seats hot and, therefore, discouraged use. One participant shared, “I use it at night due to heat from the sun. If you sit on it at day time, one may end up with a burn” (Dinka male). Likewise, a Somali woman asserted, “When the sun is too hot, they can’t use it.” In addition to discouraging use, direct sun exposure caused an unpleasant odor and may have reduced the effectiveness of ash as a means of odor control.

#### Group-specific challenges

Beyond those noted above, a few challenges were specific to each language group. These challenges often served as the basis for the user’s preference, especially for men, for the old or new system.

Somali Participants: Urine and Liquid Diversion. Somali users heavily critiqued the urine and liquid-diversion feature. This theme was pronounced in all of the Somali post-implementation FGDs. Both men and women had difficulty using the feature. Somali men and women practice anal washing as part of their belief system. According to the participants, having separate holes for the liquid contents and feces made this practice extremely challenging because it was difficult to direct the anal washing water into the urine and liquid hole without splashing into the other and, ultimately, discouraged frequent use. One Somali male indicated, “[B]ecause we are Muslims, [we] can’t keep moving from one hole to another. The previous latrine I used to visit 3–5 times in a day, unlike this one, that I can only visit 1–2 times in a day.” Additionally, several women had been subjected to female genital mutilation (FGM), which further limited their ability to use this feature. One Somali woman shared, “…especially for those who underwent female genital mutilation. It is hard for them to separate the two” (Somali female).

Dinka: Number of Users and User Demographics. Dinka households tended to have a greater number of users per toilet, with an average of 16 (range 7–40), presenting a challenge for some Dinka participants. As noted in the Dinka male FGD, Too many users made toilet cleaning and maintenance more challenging, especially when several of the household members were young children. Dinka males suggested that young children were more prone to misuse, thereby making the toilets dirtier and increasing the likelihood that the children would come into contact with human waste.

Dinka: Toilet Maintenance and Upkeep. Likely resulting from the high number of household and non-household users and a lack of cleaning supplies, Dinka participants indicated that toilet maintenance, specifically cleaning the toilet, was cumbersome and challenging. This influenced overall perceptions of the new system, particularly among Dinka males. “I do not see anything good with this new [toilet] because if it is not cleaned, it does not operate well” (Dinka male). While Dinka women also noted several challenges associated with toilet maintenance, these were most often associated with securing necessary supplies such as cleaning disinfectant and ashes—used to reduce smell.

#### Preference for new or previous system

Both Dinka and Somali women reported a preference for the new system. Dinka women preferred having the waste collected, which reduced the presence of cockroaches. “I like the new type of [toilet]. I like it because when it gets filled up, it is emptied, unlike the old type that spreads cockroaches” (Dinka female). Similarly, Somali women preferred the waste collection of the new system. “We prefer them because after 3 days, the waste is being collected” (Somali female), noting a decrease in smell when compared to the previous latrines. When Somali women noted a preference for the old system, it was because they did not prefer urine and liquid diversion, at times due to FGM. However, in general, women described the new toilets as cleaner and more hygienic.

Unlike the women, the men’s opinions were more mixed. Dinka men preferred to sit on a toilet rather than squat over a latrine. Further, Dinka men indicated that the new toilets were easier to install (compared to digging a pit) and reduced foul odors and the presence of insects. When a preference for the new toilet was noted, it was qualified by the provision of cleaning supplies. One Dinka male noted, “It is good when emptied and washed, so that it smells good. It is comfortable to use then.”

Like Dinka men, Somali men were divided on their preference for the new system. Men who preferred the new toilets often referenced the sitting design. Similar to Somali women, the most salient critique of the new toilets was urine and liquid diversion, which made anal washing, an important religious practice, more difficult.

#### Thoughts on scaling-up the intervention

When asked whether the intervention should be implemented in the newest area of the camp, Dinka men, Dinka women, and Somali women supported scaling-up the intervention, referencing the easy installation, the challenges of the old system, and the appropriateness of the new system for special populations. In contrast, Somali male perspectives were more varied. Those in support of the scale-up at times emphasized the need for redesign (removal of the urine and liquid diversion feature) and focused on the speed at which the new system could be installed compared to the previous latrines. Others were opposed to scale-up in new areas of the camp, but were in favor of scale-up in older areas of the camp, and noted several reasons, including: ample land in newer areas that could be used for pit latrines.

#### Thoughts on briquette use

Most participants were open to briquette use. Some mentioned seeing this type of technology previously. Rare apprehensions regarding use centered on concerns about smell and safety, but did not appear to significantly deter interest.

## Discussion

While the urgent need for improved sanitation in refugee camp settings is well documented, [5][24][25] sanitation solutions are rarely presented that deviate from traditional pit latrine-based approaches. Moreover, despite recommendations to include refugees—particularly women and individuals with special needs—into sanitation planning and implementation[26][27], there is a lack of available evidence in the literature to suggest that this has been widely accomplished. For example, research based in Kakuma highlighting the challenges disabled residents experience when trying to facilitate and navigate pit latrine use, stopped short of recommending alternative sanitation options.[25] Sanitation considerations are further compounded by the cultural and religious diversity found in many camp settings. Documentation of poor sanitation, including open defecation and overcrowding, in camps with large number of Dinka and Somali residents are available; however, no specific statistics on sanitation preferences among these groups are offered. [28][29][30][5][7] However, as highlighted in the findings from this study, cultural and religious differences can greatly influence sanitation needs.

Based in Kakuma refugee camp and using mixed-methods that centered on understanding resident experiences and needs, our objective was to design an alternative novel sanitation system, pilot it with a small sample of residents, and assess their feedback on the use and appropriateness of the new system. Camp sanitation is currently based on pit latrine use. Though common, pit latrines burden already scarce resources and, due to overcrowding, may pose a health risk to users.

Residents were very aware of the sanitation challenges present in the camp and emphasized a need for alternative sanitation solutions. Throughout the pre-implementation FGDs, residents expressed their concerns about the camp’s current sanitation system and outlined a number of challenges, including insufficient numbers of latrines, foul odors, risks to children, poor latrine construction, pests, and the need for more cleaning supplies. Overcrowding and the continued practice of open defecation were also emphasized and served to amplify residents’ concerns.

Dinka participants frequently reported practicing open defecation before coming to the camp as a matter of lifestyle and tradition, and lack of access to improved sanitation may influence the continuation of this practice by some camp residents. Open defecation increases the risk of diarrheal disease transmission, whereas access to private, improved sanitation facilities can reduce the risk of exposure to the others’ feces, and is thus, safer.[31][32][33]

In 2015, an estimated 638 million people relied on shared sanitation facilities (398 million in urban areas and 240 million in rural areas), which are classified as unimproved by virtue of the fact that they are shared, even if otherwise improved.[1] Dinka and Somali participants reported sharing their latrines with many other users. (In accordance with SPHERE standards, NRC maintains there was one latrine for every 20–25 camp residents.) Even with household toilets, some Dinka participants, who, on average, reported larger household sizes, remained frustrated with the number of users, specifically when the toilet was shared with young children.

Overall, participants collectively preferred the new toilet, with a higher level of preference reported among the Dinka population. Many were satisfied with the new system because they thought the sitting design was more ergonomic, there was reduced odor and fewer pests compared to the current system, and it was more applicable to vulnerable populations such as elderly or disabled people.

Participants were also pleased with the waste collection and treatment process. The new system, whereby sanitation is provided as a service, provided jobs and training to waste collection representatives and waste treatment and reuse staff, demonstrating potential promise for sustainable microenterprise and scale-up in the future. Additionally, transforming waste into a usable commodity could potentially supplement fuel shortages in the refugee camp and provide an affordable fuel alternative in other areas. New kinds of sanitation technology will require collaboration with various stakeholders, including public health experts, and where necessary, seeking approvals from regulatory bodies, such as National Environment Management Authority, and ensuring safe handling and transportation of waste. While the briquettes made during the study were not distributed to camp residents, most residents expressed a willingness to use the briquettes for cooking if they were made available. The microbiologic analysis of the briquettes by the Kenya Bureau of Standards would further help in creating awareness and promoting user acceptability of the briquettes to the beneficiaries, clearing potential doubts regarding the safety and usage.

This 6-week pilot had several limitations. Most notably, the duration of the study limited our ability to determine whether the piloted sanitation management system remained acceptable and appropriate over time. Future efforts should incorporate not only the redesign of the toilets based on community feedback but should also occur over a more extended time period to better determine long-term viability and user acceptability. Additionally, due to resource and time constraints, all toilet designs were not piloted in all study households, limiting our ability to speak directly to the benefits or challenges associated with specific toilet types. Further, the sample size was small and all nationalities in the camp were not represented. Only 32 households were provided toilets, and subsequently, only a small number of participants were included in the bi-weekly surveys and post-implementation FGDs. This, along with the sampling method selected, limited the generalizability of the study findings. Despite these limitations, we were able to use the data collected to design, implement, and pilot a novel sanitation system. Data from the current pilot are essential to improving the design of the toilets ensuring that the intervention is practical and appropriate for use in field settings.

## Conclusions

Ensuring access to improved sanitation remains a pressing global issue.[2] The use of communal pit latrines in protracted refugee camp settings can increase the risk of infectious disease transmission.[4] Increasing access to improved sanitation can reduce diarrheal disease morbidity and improve quality of life in refugee settings.[34] Collaborators worked together in Kakuma refugee camp to pilot a sanitation management system that incorporated service-based waste removal, treatment, and reuse. Overall, the system was well-received; however, the urine and liquid diversion feature presented a challenge for many Somali users. This feature will need to be re-evaluated or refined to increase user acceptance among certain groups. Using residents’ input to inform the development of sanitation solutions can increase user acceptability, thereby providing opportunities to design culturally appropriate sanitation systems based on specific community-defined needs. Further, with continued prototype modification and further evaluation, alternative low-cost sanitation systems, such as the one piloted in Kakuma, may have much broader applications in underserved rural and urban populations.

### Disclaimer

The findings and conclusions in this report are those of the authors and do not necessarily represent the official position of the U.S. Centers for Disease Control and Prevention.
