# Correction: Sanitation practices and perceptions in Kakuma refugee camp, Kenya: Comparing the status quo with a novel service-based approach

**DOI:** 10.1371/journal.pone.0190129

**Published:** 2017-12-19

**Authors:** Raymond Nyoka, Andrew M. Foote, Emily Woods, Hana Lokey, Ciara E. O’Reilly, Fred Magumba, Patrick Okello, Eric D. Mintz, Nina Marano, Jamae F. Morris

The second author’s name is spelled incorrectly. The correct name is: Andrew M. Foote. The correct citation is: Nyoka R, Foote AM, Woods E, Lokey H, O’Reilly CE, Magumba F, et al. (2017) Sanitation practices and perceptions in Kakuma refugee camp, Kenya: Comparing the status quo with a novel service-based approach. PLoS ONE 12(7): e0180864. https://doi.org/10.1371/journal.pone.0180864
